# DOES PARTIAL MEDIAL KNEE ARTHROPLATIES HAVE BETTER RESULTS THAN TOTAL ONES?

**DOI:** 10.1590/1413-785220233102e262186

**Published:** 2023-06-09

**Authors:** JOÃO PAULO FERNANDES GUERREIRO, VITORIA KAROLINE JUSTINO DOS SANTOS, LUCAS BECKERT MATZ, LUCAS DELFINO PEDROLLO, VITOR HENRIQUE JUSTINO DOS SANTOS, ALEXANDRE OLIVEIRA QUEIROZ, PAULO ROBERTO BIGNARDI, MARCUS VINICIUS DANIELI

**Affiliations:** 1. Pontifícia Universidade Católica do Paraná (PUCPR), Schoolof Medicine at PUCPR- Londrina Campus, Londrina, PR, Brazil.; 2. Hospital Evangélico de Londrina, Londrina, PR, Brazil.; 3. Hospital de Ortopedia UNIORT.E, Londrina, PR, Brazil.

**Keywords:** Knee, Arthroplasty, Osteoarthritis, Arthroplasty, replacement, knee, Joelho, Artroplastia, Osteoartrite, Artroplastia do Joelho

## Abstract

**Objective:**

Compare the results of medial unicompartmental knee arthroplasty (UKA) using a mobile platform and total knee arthroplasty (TKA) in patients with isolated medial osteoarthritis.

**Methods:**

Retrospectivecross-sectional study. Preoperative radiographs of 602 patientswho underwent knee arthroplastybetween February 2017 and February 2020 were evaluated. Isolated medial osteoarthritis was found in 125 patients. Of these, 57 underwent UKA and 68 TKA. With chart analysis and telephone interviews, we compared patients’ clinical outcomes and degree of satisfaction. The statistical analysis used a confidence level of 5%.

**Results:**

The group of UKA patients obtained 65.8% of favorable results against 79.1% of those undergoing TKA in the function questionnaire (p<0.0001). The complication rate was statistically similar between the groups(p>0.5). Most patients were satisfied or very satisfied in both groups (88.6% of UKA and 91.2% of TKA) (p>0.999).

**Conclusion:**

Patients submitted to UKA or TKA have presented the same degree of satisfaction and rate of postoperative complications when comparing patients with isolated medial osteoarthritis. UKA patients had less favorable results onthe clinical functional questionnaire than patients undergoing total arthroplasty. Level Of Evidence III;Retrospective Study.

## RESUMO

**Objetivo:**

Comparar os resultados da artroplastia unicompartimental do joelho (UKA) medial com plataforma móvel e artroplastia total do joelho (TKA) em pacientes com osteoartrose medial isolada.

**Métodos:**

Estudo transversal retrospectivo. Foram avaliadas radiografias pré-operatórias de 602 pacientes submetidos à artroplastia de joelho entre fevereiro de 2017 e fevereiro de 2020. A osteoartrose medial isolada foi encontrada em 125 pacientes, destes em 57 haviam sido submetidos a UKA e 68 TKA. Com análise de prontuários e entrevistas telefônicas comparamos os resultados clínicos e o grau de satisfação dos pacientes. A análise estatística utilizou nível de confiança de 5%.

**Resultados:**

O grupo de pacientes com UKA obteve 65,8% de resultados favoráveis contra 79,1% daqueles submetidos à TKA no questionário de função (p<0,0001). A taxa de complicações foi estatisticamente semelhante entre os grupos (p>0,5). A maioria dos pacientes estava satisfeita ou muito satisfeita em ambos os grupos (88,6% de UKA e 91,2% de TKA) (p>0,999).

**Conclusão:**

Pacientes submetidos a UKA ou TKA apresentaram o mesmo grau de satisfação e taxa de complicações pós-operatórias quando comparados pacientes com osteoartrite medial isolada. Os pacientes da UKA apresentaram resultados menos favoráveis ao questionário clínico funcional do que os pacientes submetidos à artroplastia total. Nível de Evidência III; Estudo Retrospectivo.

## INTRODUCTION

Unicompartmental Knee Arthroplasty (UKA) is an alternative to total knee arthroplasty (TKA).^1^ Studies demonstrated that medial UKA is associated with a shorter hospital stay, lower infection rate and better range of motion, compared to TKA.^2 - 4^

However, some studies have shown that UKA has a higher rate of revisions in up to 10 years compared to TKA.^4 - 6^ This higher rate of reoperations relates directly to the surgeon’s experience with UKA through the number of surgeries performed annually, as 80% of knee surgeons perform less than 10 UKAs per year.^7^ The TOPKAT randomized clinical trial found similar rates of surgical revisions in patients undergoing UKA and TKA.^8^ A systematic review of over 8000 patients who underwent Oxford® unicompartmental medial arthroplasty demonstrated that the rate of revisions alone was higher in UKA. However, if considered all surgical reapproaches, this number was higher in TKA.^6^

Some individuals with an indication for unicompartmental medial prosthesis often still undergo total prosthesis in our country because they are still not available in some centers, and some surgeons are still unaware of the indications and techniques.^9^

In this study, we compared the clinical outcomes and degree of satisfaction of patients with isolated medial compartment osteoarthritis of the knee who underwent medial knee replacement with the Oxford Mobile Platform or TKA in the same period with at least six months of postoperative follow-up.We hypothesized that there were better clinical outcomes and a higher degree of satisfaction in patients undergoing UKA.

## METHODS

The study was approved by the Ethics and Research Committee of the Institution (CAAE: 31747020.8.0000.0020).

A retrospective cross-sectional study with chart analysis and telephone interviews was conductedto compare the clinical outcomes and degree of satisfaction. Patients who underwent Oxford® mobile bearing medial unicompartmental arthroplasty or total knee arthroplasty by our knee surgery group between February 2017 and February 2020 were selected.

Inclusion criteria were patients with preoperative radiographs that fit the radiographic criteriafor medial unicompartmentalarthroplasty,^10 - 11^ undergoing total or medial unicompartmental arthroplasty with a follow-up of at least six months, with complete medical records and the possibility of phone contact. The analysis of all preoperative radiographs and medical records excluded patients with less than 6 months of follow-up, patients older than 75, and younger than 50 years of age, to form two homogeneous groups concerning age and follow-up time.

The following information was searched in the medical records: name, birth date, date of surgery, side (left or right knee), kind ofsurgery (UKA or TKA), and complications. After telephone contact, reading and approval of the consent form, the patient answered a questionnaire that consisted of the following questions: degree of satisfaction with the surgery (very dissatisfied, somewhat dissatisfied, somewhat satisfied, satisfied or very satisfied), whether he/she would do the surgery again (yes or no), whether he/she would recommend the same surgery to an another patient (yes or no), the length of hospitalization (number of days), if there was a readmission in the first 30 days after surgery, if there was deep vein thrombosis in the first 30 days after surgery, if there was an acute myocardial infarction in the first 30 days after surgery, if there was a stroke in the first 30 days after surgery, if died in the first 30 days after surgery, if had wound problems, if have undergone new surgery (debridement, surgery for arthrofibrosis, peri-prosthetic fracture, or revision surgery), if have had revision surgery, what was the cause (stiffness, infection, aseptic loosening, peri-prosthetic fracture or persistent pain after surgery), and if have any symptoms in the knee (yes or no), if yes what symptoms are present: any walking difficulty (yes or no), can support body weight on the operated leg (yes or no), any difficulty using stairs (yes or no), any difficulty squatting (yes or no), usually have swelling in the knee (yes or no), can bend the knee to 90 degrees of flexion (yes or no), does it bother with any crepitus or “noise” when you move the knee (yes or no).

The Chi-square (x2) or Fisher’s Exact test were used in statistical analysis to evaluate the qualitative variables. For quantitative variables, Shapiro-Wilk test was firstly applied to analyze normality. Subsequently, the Mann-Whitney test for non-normal data and the t test for variables with Gaussian distribution. The results were analyzed using GraphPad Prism8 software (GraphPad Software Inc., La Jolla, CA, USA), with a 5% confidence level established for all tests applied.

## RESULTS

**Figure 1:**
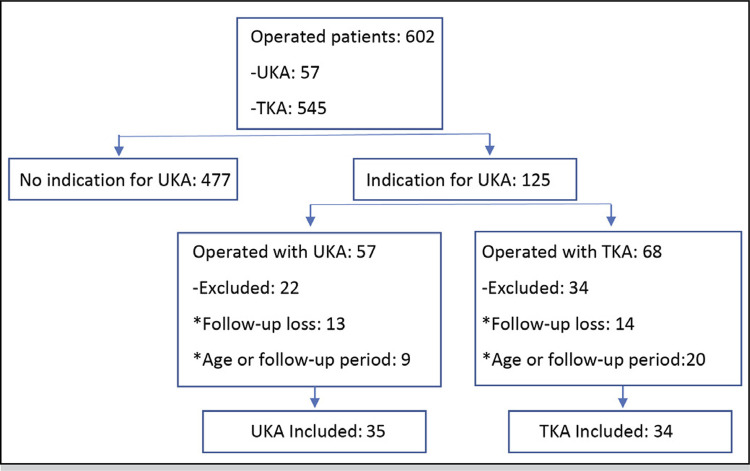
Study flowchart.

A total of 602 radiographs of patients undergoing TKA (545) and UKA (57) between February 2017 and February 2020 were evaluated. After applying the radiographic criteria,^10 - 11^ 477 patients were excluded. Of the 125 included, 57 had been submitted to UKA and 68 to TKA.There were13 patients of the UKA group that lost follow-up and 14 patients of the TKA group. There were 9 patients in the UKA group and 20 in the TKA outside the limits of maximum and minimum age and follow-up time. ( Figure 1 )

The two groups were homogeneous in terms of age, sex, and follow-up time. ( Table 1 )

**Table 1:** Demographic data.

	UKA	TKA	P Value
Age (years)	64,1 ± 9,6	64,1 ± 6,9	0,998
Male n (%)	9 (25,7)	9 (26,5)	>0.999
Follow-up time (years)	25.8 ± 10,7	30,2 ± 9,7	0,072

Regarding the results of the clinical functional questionnaire, 65.8% of the answers from the patients submitted to UKA were favorable, against 79.1% of those submitted to TKA.Such data was statistically significant (p< 0.0001).( Table 2 )

**Table 2:** Clinical functional questionnaire.

	UKA	TKA	OR (CI 95%)	P Value
Favorable results	65.8%	79,1%	0,51 (0,36 - 0,72)*	<0,0001

* As a reference to the UKA; If the reference is the TKA, the OR is = 1.97 (1.39 - 2.78).

There was no difference between groups regarding the satisfaction rate (P>0.999) (88.6% in the UKA and 91.2% in the TKA). ( Table 3 )

**Table 3:** Satisfaction rate.

	UKA (n=35)	TKA (n=34)	OR (CI 95%)	P Value
Satisfied or very satisfied	31 (88,6%)	31 (91,2%)	0,75 (0,18 - 3,00)*	>0.999

* Fischer’s exact test. OR concerning UKA; if reference is to TKA, it gets OR=1.33 (0.33 - 5.61).

Regarding the complications rate there was no statistical difference between groups(p>0.5). ( Table 4 )

**Table 4:** Complications.

	UKA	TKA	OR (CI 95%)	P Value
Incidence of complications, n(%)	9 (25,7)	12 (35,3)	0,63 (0,22 - 1,70)	0.440
Superficial and deep infections, n(%)	3 (8,6)	5 (14,7)	0,54 (0,14 - 2,38)*	0.477
Readmission within 30 days, n(%)	1 (2,9)	3 (8,8)	0,30 (0,02 - 2,16)*	0.356
Revision surgery (aseptic loosening), n(%)	3 (8,6)	1 (2,9%)	3,09 (0,43 - 41,23)*	0,613

* Fischer’s exact test.

## DISCUSSION

This study showed that there was no significant difference between TKA and UKA concerning satisfaction, in patients with medial osteoarthritis.

The “TOPKAT” randomized clinical trial^8^ showed a trend toward patients being more satisfied with the unicompartmentalarthroplasty, but no statistical difference between the groups. We found a trend in favor of the total knee arthroplasty but no statistical difference between groups.

There was also no statistical difference regarding complications.Literature presents extensive retrospective studies demonstrating fewer complications in medial UKA and a higher number of revisions when compared to the TKA.^4 , 5 , 7^ However, these studies compare groups of patients undergoing total and unicompartmentalarthroplasty without considering the degree of preoperative impairment. Therefore, in our study, we compared two homogeneous groups (medial focal osteoarthritis).This was possible because the unicompartmental medial prosthesis was not yet routinely performed by all surgeons in the group. Many patients were still unaware of this option, while some health insurances did not authorize this type of implant when the surgeries were performed. Therefore, many patients that would have a precise indication for medial UKA underwent total knee arthroplasty. The “TOPKAT” randomized clinical trial^8^ also compared the results of unicompartmental and total knee arthroplasty in patients who had similar preoperative impairment degrees. It showed similar number of complications.In the same study,^8^ the authors discuss that the higher numbers of complications shown in previous large retrospective studies^3 - 5^ could be a consequence of the lack of experience of the surgeon where the unicompartmental surgery was performed. The results of our study did not confirm this thesis completely, although there was a higher number, but no statistical difference, of aseptic loosening in the unicompartmental prosthesis group.The follow-up of a higher number of patients may show a statistically significant difference.

The rate of favorable responses found about function and symptoms in the knee was higher in patients submitted to UKA compared to those submitted to TKA. This data differs from that found in the literature^8^ and we believe that it may have been influenced by our team’s less experience in performing unicompartmental arthroplasty compared to total arthroplasties at the time of the surgeries. Another fact that may have influenced the answers to the clinical and functional questionnaire is the tendency to create higher patient expectations of clinical and functional results with UKA because it is a less invasive and more preservative surgery when compared to TKA.

We found several limitations in our study. First, the average follow-up time was approximately two years, whereas we predicted durability of more than ten years in most arthroplasty cases.Second, the number of patients is limited for a retrospective cohort on this topic. Third, our patients are from a single center which limits the representativeness of the population. Fourth, we have no preoperative clinical or functional evaluation. Fifth, we did not do any objective functional tests on the patients, just a simplified, non-standardized questionnaire about clinical symptoms and signs. This may make it difficult to compare our results with other studies.

## CONCLUSION

Patients with isolated medial osteoarthritis who underwent unicompartmentalmedial mobile bearing arthroplasty had the same degree of satisfaction and postoperative complication rate as patients who underwent total knee arthroplasty. These patients had less favorable clinical functional questionnaire answers than patients who had undergone TKA.
